# Cancer: UV Protection from Plants

**Published:** 2008-06

**Authors:** M. Nathaniel Mead

Excessive exposure to the sun’s ultraviolet (UV) rays can exact a heavy toll on skin health, resulting in an increased risk of skin cancers as well as other telltale signs of photoaging. In the United States alone, more than 1 million cases of skin cancer are diagnosed each year, accounting for almost 40% of all new cancer cases, according to the National Cancer Institute. Thus, the search continues for better ways to prevent the deleterious effects of too much sun. One approach, photochemoprotection, uses pharmacologically active plant-derived compounds, administered either orally or topically, to prevent carcinogenic sun damage.

A compelling feature of photochemoprevention is its focus on specific aspects of skin cancer biology. “Phytochemicals can be employed to intervene at the initiation, promotion, or progression stages of the multistage cancer process,” says Farrukh Afaq, a dermatology researcher at the University of Wisconsin–Madison. “Since multiple pathways are involved in [the] photocarcinogenic response, a mixture of several phytochemicals working through different cell signaling pathways or other mechanisms could be an effective strategy.”

A review by Afaq and colleagues in the March–April 2008 issue of *Photochemistry and Photobiology* summarizes the photochemoprotection strategies that show the most promise, among them green tea polyphenols (GTPs), grape seed proanthocyanidins (GSPs), resveratrol from grapes, silymarin from milk thistle, curcumin from turmeric, beta-carotene, and extracts of pomegranate fruit. Afaq notes that many of these compounds have been shown to target the NF-κB, AP-1, MAPK, and PI3K/AKT pathways, all of which are involved in photocarcinogenesis and the progression of skin cancers.

“Along with antioxidant, anti-inflammatory, and DNA repair effects, a number of these agents also have immune-modulating properties,” says Santosh Katiyar, a dermatology professor at the University of Alabama at Birmingham who was not a part of the study. “This is important, since chronic exposure to solar UV radiation suppresses immune reactions, and since such suppression has been implicated in the development of skin cancer.”

Under the guidance of UW–Madison cancer researcher Hasan Mukhtar, the group has launched a program to define the cancer-curbing potential of phytochemicals and establish their mechanisms of action. “These phytochemicals essentially function in two ways,” says Mukhtar. “The first way is to have the ability to scavenge highly damaging and reactive free radicals that are formed when the skin is exposed to solar UV rays. The second is through their effects on signal transduction molecules that are induced in the skin in response to UV rays.”

Most of the evidence to date has come from laboratory data, mainly for reasons of cost and practicality. “Evidence from human trials continues to be quite limited, in large part because of the long period of photocarcinogenesis in humans,” says Stephen Hsu, a molecular medicine professor at the Medical College of Georgia in Augusta. “These trials may take ten or more years in comparison to rodent studies, which can be completed in a matter of months.” In addition, he says, the bioavailability of GTPs and GSPs in their original forms is very low, whether used orally or topically, so high doses had to be used in the animal studies to achieve the desired efficacy. The Wisconsin group notes in their review that combinations of antioxidant phytochemicals may therefore be necessary to achieve the desired level of photochemoprevention.

“Some skin care products already contain phytochemicals,” says Afaq. “In conjunction with other sun-safe measures, use of these products may be an effective approach for reducing [UV-mediated] photodamage, inflammatory responses, photoaging, and skin cancer in humans.” Afaq and Mukhtar assert that individuals can modify their dietary habits in combination with targeted use of skin care products and botanical antioxidants, perhaps eventually enabling people to enjoy the sun’s health benefits [see “Benefits of Sunlight: A Bright Spot for Human Health,” *EHP* 116:A160–167 (2008)] without the dermatologic consequences.

